# Modeling and solving the parallel mixed-flow remanufacturing disassembly line balancing problem for multi-variety products

**DOI:** 10.1038/s41598-022-19783-4

**Published:** 2022-09-13

**Authors:** Gang Yu, Xiufen Zhang, Wei Meng

**Affiliations:** 1 Inner Mongolia Technical College of Mechanics and Electrics, Hohhot, 010070 People’s Republic of China; 2 The College of Mechanical Engineering, Inner Mongolia University of Technology, Hohhot, 010051 People’s Republic of China; 3 Design and Research Institute of Shaanxi, Huanghe Group Co. LTD, Weinan, 710043 People’s Republic of China

**Keywords:** Engineering, Mathematics and computing

## Abstract

The types and numbers of components in end-of-life (EOL) products are often uncertain during remanufacturing, leading to low disassembly efficiencies for traditional remanufacturing disassembly lines. To address this problem, a parallel mixed-flow workstation layout was designed, and a novel parallel mixed remanufacturing disassembly line balancing optimization method for multi-variety products was proposed. A mixed-flow product disassembly task hierarchical assignment matrix was constructed to perform disassembly task allocations for similar components. Furthermore, a parallel mixed-flow remanufacturing disassembly line balancing (PMRDLB) optimization model was developed with the optimization objectives of minimizing the number of workstations, the disassembly line balancing rate, and the remanufacturing value indexes of the components. Furthermore, the multi-objective non-dominated genetic optimization method (NSGA-III) was improved, in which a chromosome construction method, based on the parallel mixed-flow disassembly task allocation matrix, was proposed to conduct mapping between the chromosomes and the PMRDLB model. In addition, non-dominated solution sorting was performed based on a Pareto hierarchy, which increased the searching rate of the algorithm during optimization. Finally, a case study verified the effectiveness and feasibility of the proposed method.

## Introduction

Remanufacturing is a profitable means of recovering end-of-life (EOL) products. Disassembly is the key step in obtaining the remanufacturing cores. The remanufacturing disassembly line balancing problem (RDLBP) focuses on obtaining an optimal disassembly line configuration scheme with reasonable task allocations, balanced workstation operations, a high disassembly efficiency, and a low cost, thereby reducing the remanufacturing costs^1,2^.

Three classical disassembly line layouts exist: straight line, U-type, and parallel-type layouts. Therefore, the classical disassembly line balancing problem (DLBP) includes single-type linear bilateral disassembly line balancing^3^, incomplete single-type linear disassembly line balancing^4^, U-type disassembly line balancing^5,6^, and single-type parallel disassembly line balancing^7–10^.For example, to increase the disassembly efficiency for large products, a two-sided layout was introduced, and a mathematical model for a stochastic two-sided partial DLBP with multiple objectives, multiple constraints, and uncertainty was constructed and resolved based on the multi-objective discrete flower pollination algorithm^3^. Li et al.^4^ developed an incomplete single-type linear disassembly line balancing model and proposed the variable neighborhood particle swarm optimization algorithm. A profit-oriented U-shaped partial DLBP was proposed and solved using a discrete cuckoo search algorithm^5^. To improve the disassembly line production efficiency and reduce the production cost, the parallel disassembly line balancing problem was studied^7,8^. Zhu et al.^10^ developed a mathematical model for a multi-objective locally parallel disassembly line balancing problem and solved the problem using the hybrid group neighborhood search algorithm.

Unfortunately, the types and numbers of components are often uncertain for EOL products during remanufacturing disassembly, which causes significant challenges for batch disassembly. The same types of products must be identified and rearranged for the existing disassembly lines, which is a complicated and low-efficiency process. Thus, much attention has been paid to parallel mixed-flow disassembly lines since they can significantly improve disassembly efficiency^2,8^.Therefore, Agrawal and Tiwari^11^ introduced the mixed product disassembly line concept and constructed a random mixed U-shaped disassembly line model. Model resolution was difficult using traditional methods. Later, Xia et al.^12^ selected multiple products as the mixed products based on their structural similarities, developed a mixed disassembly line model under a random working environment, and solved the problem by adopting the adaptive simulated annealing genetic algorithm. Fang et al.^13^ constructed a multi-robot hybrid disassembly line model and applied the evolutionary simulated annealing algorithm to obtain the optimal solution. Zeng et al.^14^ constructed a multi-objective bucket-chain disassembly line model and proposed a multi-objective discrete flower pollination algorithm to solve the problem.

All of these researchers solved the mixed-flow disassembly line balancing problem by assuming multi-variety products as the mixed products. However, it becomes more difficult to construct a mixed product model as the products’ types and complexities increase. To address this problem, a hierarchical parallel workstation layout is designed for the first time in this paper, furthermore, a parallel mixed-flow remanufacturing disassembly line balancing (PMRDLB) optimization model was constructed for multi-variety products. The main features of the proposed model are outlined as follows:

1. It not only made reasonable use of space but also improved the efficiency of parallel mixed-flow disassembly for multi-variety products with uncertain characteristics in remanufacturing disassembly lines.
2. It overcame the difficulties of model construction and low computational efficiency caused by the traditional mixed disassembly line in which multiple products were regarded as a single imaginary mixed product.

The DLBP can be solved primarily by mathematical programming, heuristic optimization, or meta-heuristic optimization. Mathematical programming produces high solution precision, but it is only suitable for solving small-scale disassembly line balancing tasks^15–18^. Heuristic methods can solve large-scale disassembly line problems, but their solutions will be limited to local optima^19,20^. Meta-heuristic methods are the mainstream algorithms used to solve the DLBP; they include the multi-objective genetic algorithm (GA), the multi-objective genetic annealing algorithm, and the artificial fish swarm algorithm, among others^21–24^. These methods are often combined with multi-criteria decision technology when solving the problem^25^. Among them, the GA is robust and suitable for parallel computing and has been widely used for solving the DLBP^26,27^. Therefore, in this paper, which focuses on the layout characteristics of a multi-variety parallel mixed-flow remanufacturing disassembly line, an improved multi-objective non-dominated sorting genetic optimization method (Improved NSGA-III) is proposed to solve the PMRDLB problem.

## Methods

### Problem description

**Figure 1:**
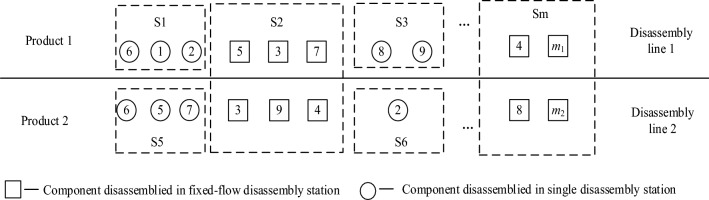
Parallel mixed-flow remanufacturing disassembly line layout diagram.

There are different types of EOL products for remanufacturing with uncertain quantities. To achieve a reasonable allocation of disassembly tasks for different types of products, this paper proposes a parallel mixed-flow disassembly line layout, as shown in Fig. 1.

If there were two kinds of EOL products to be disassembled and the number of components was uncertain, two disassembly lines were required. Parallel stations were arranged on each disassembly line, such as stations S1 and S3 in Fig. 1. The two adjacent disassembly lines had mixed-flow disassembly stations, such as stations S2 and Sm. All disassembly tasks were assigned to *N* workstations according to the determined beat time, *CT*.

The parallel mixed-flow remanufacturing disassembly line balancing problem focused on attaining a reasonable allocation of disassembly tasks in the layout shown in Fig. 1 to minimize the number of disassembly stations, prioritize the disassembly of components with high remanufacturing values and hazardous material properties, and rationally utilize the factory space of the enterprise.

To simplify the problem, three assumptions were made:

1. The disassembly time and remanufacturing value of each component were known, and all disassembly tasks were independent.
2. A disassembly task could not be interrupted.
3. The same disassembly task could not be assigned to multiple stations at the same time.

### Judgment conditions for the mixed-flow disassembly of multi-variety products

Similarities and differences exist in the physical, material, and geometrical structures of various types of EOL products. Only products with certain similarities can be disassembled using a parallel mixed-flow disassembly line^12^. Therefore, it was necessary to determine the degree of product similarity.

It was assumed that the two disassembly task sets for the EOL products were

${\mathbf{P}}_{1}=\left\{{a}_{1}^{1},{a}_{2}^{1},{a}_{3}^{1},\cdots ,{a}_{m1}^{1}\right\}$ and ${\mathbf{P}}_{2}=\left\{{a}_{1}^{2},{a}_{2}^{2},{a}_{3}^{2},\cdots ,{a}_{m2}^{2}\right\}$. The similar components set was

$\mathbf{S}=\bigcap \left\{{a}_{1}^{1},{a}_{1}^{2},{a}_{2}^{1},{a}_{2}^{2},{a}_{3}^{1},{a}_{3}^{2}\cdots ,{a}_{m1}^{1},{a}_{m2}^{2}\right\}$, and the total components set was

${\mathrm{P}}_{1}{\mathrm{P}}_{2}=\bigcup \left\{{a}_{1}^{1},{a}_{1}^{2},{a}_{2}^{1},{a}_{2}^{2},{a}_{3}^{1},{a}_{3}^{2}\cdots ,{a}_{m1}^{1},{a}_{m2}^{2}\right\}$. Thus, the similarity degree between the two products could be defined as follows:

1 $$ \lambda_{pro} = \frac{{ \cap \left\{ {a_{1}^{1} ,a_{1}^{2} ,a_{2}^{1} ,a_{2}^{2} ,a_{3}^{1} ,a_{3}^{2} \cdots ,a_{m1}^{1} ,a_{m2}^{2} } \right\}}}{{ \cup \left\{ {a_{1}^{1} ,a_{1}^{2} ,a_{2}^{1} ,a_{2}^{2} ,a_{3}^{1} ,a_{3}^{2} \cdots ,a_{m1}^{1} ,a_{m2}^{2} } \right\}}}\frac{{\mathbf{S}}}{{{\mathbf{P}}_{1} {\mathbf{P}}_{2} }}\quad 0 \le \lambda_{pro} \le 1 $$

where *m*1 and *m*2 are the numbers of components in the two EOL products to be disassembled. The larger the similarity degree was, the greater was the similarity between the components in geometrical, physical, and material aspects, among others. When ${\lambda }_{pro}=0$, there is no similarity between the components of the two EOL products. Empirically then, when ${\lambda }_{pro}\ge 0.7$, the mixed-flow disassembly can be performed^12^.

### Mathematical model for the parallel mixed-flow remanufacturing disassembly line balancing (PMRDLB) problem

A mathematical model for the PMRDLB problem was developed based on the parallel mixed-flow remanufacturing disassembly line layout shown in Fig. 1. For clarification, the symbols utilized in the mathematical model are defined in Table 1, and the acronyms in this paper are listed in Table 2.

**Table 1:** Symbol definitions.

Symbol	Illustration
*mk*	Number of the *kth* EOL product’s disassembly tasks
*N*	Number of disassembly stations
*CT*	Disassembly line beat time
*t*_*il*_	The disassembly time of the task *i* on the *lth* disassembly line
*x*_*ilr*_	Station task allocation coefficient, when the disassembly task *i* on the *l*th disassembly line is assigned to the *rth* station, it equals 1, and otherwise, it equals 0
*P*_*il*_	Remanufacturing value of the components disassembled in the disassembly task *i* of the *lth* disassembly line, if the component has no remanufacturing value, it equals 0, and otherwise, it equals 1
*S*_*r*_	Disassemble task set in station *r*
*L*_*il*_	The position of component in the disassembly sequence in the *ith* disassembly task of the *lth* disassembly line
*S*_*ij*_	Task *i* takes precedence over task *j*
${\mathbf{P}}_{mk}^{k}$	Disassembly priority mapping matrix of the EOL product *k*
${\mathbf{B}}_{mk}^{k}$	Disassembly task hazard mapping matrix of the EOL product k
*k*	Number of the disassembly lines
${\mathbf{S}}_{mk}^{k}$	The comprehensive priority relation matrix of the EOL product *k*
${\mathbf{G}}^{k}$	The disassembly task hierarchical matrix of the *kth* EOL product
**G**	The comprehensive disassembly tasks hierarchical matrix of EOL products

**Table 2:** Acronyms definitions.

Acronyms	Definitions
EOL	End-of-life
PMRDLB	Parallel mixed-flow remanufacturing disassembly line balancing
DLBP	Disassembly line balancing problem
RDLBP	Remanufacturing disassembly line balancing problem
NSGA-III	The multi-objective non-dominated genetic optimization method
GA	Genetic algorithm
MOP	Multi-objective optimization problem

One clear difference between the PMRDLB problem and the traditional DLBP is the constraint conditions. All of the products in the parallel disassembly lines should not only meet the component disassembly priority relationship requirements but should also prioritize the disassembly of toxic and harmful components to reduce secondary pollution. This type of disassembly is more complex than single-product disassembly.

The disassembly priority relationship mapping matrix for the EOL product *k* is given by

2 $${\mathbf{P}}_{mk}^{k}=\begin{array}{c}1\\ \begin{array}{c}2\\ \begin{array}{c}\vdots \\ mk\end{array}\end{array}\end{array}{\left\{\begin{array}{cc}\begin{array}{cc}{p}_{11}& {p}_{12}\\ {p}_{21}& {p}_{22}\end{array}& \begin{array}{cc}\cdots & {p}_{1mk}\\ \cdots & {p}_{2mk}\end{array}\\ \begin{array}{cc}\vdots & \vdots \\ {p}_{mk1}& {p}_{mk2}\end{array}& \begin{array}{cc}{p}_{ij}& \vdots \\ \cdots & {p}_{mkmk}\end{array}\end{array}\right\}}_{mk\times mk}$$

In Eq. (2), if task *i* is performed before task *j*, then ${p}_{ij}$ = 1; otherwise, ${p}_{ij}$ = 0.

The component hazard mapping matrix for the EOL product *k* is defined as follows:

3 $${\mathbf{B}}_{mk}^{k}=\begin{array}{c}1\\ \begin{array}{c}2\\ \begin{array}{c}\vdots \\ mk\end{array}\end{array}\end{array}{\left\{\begin{array}{cc}\begin{array}{cc}{b}_{11}& {b}_{12}\\ {b}_{21}& {b}_{22}\end{array}& \begin{array}{cc}\cdots & {b}_{1mk}\\ \cdots & {b}_{2mk}\end{array}\\ \begin{array}{cc}\vdots & \vdots \\ {b}_{mk1}& {b}_{mk2}\end{array}& \begin{array}{cc}{b}_{ij}& \vdots \\ \cdots & {b}_{mkmk}\end{array}\end{array}\right\}}_{mk\times mk}$$

In Eq. (3), if disassembly task *i* is more hazardous than task *j*, then ${b}_{ij}$ = 1; otherwise, ${b}_{ij}$ = 0.

The disassembly priority relationship for the EOL product *k* was deduced from ${\mathbf{P}}_{mk}^{k}, {\mathbf{B}}_{mk}^{k}$, and the comprehensive matrix ${\mathbf{S}}_{mk}^{k}, \mathrm{as follows}$:

4 $${\mathbf{S}}_{mk}^{k}={\mathbf{P}}_{mk}^{k}\vee {\mathbf{B}}_{mk}^{k}=\begin{array}{c}1\\ \begin{array}{c}2\\ \begin{array}{c}\vdots \\ mk\end{array}\end{array}\end{array}{\left\{\begin{array}{cc}\begin{array}{cc}{p}_{11}\vee {b}_{11}& {p}_{12}\vee {b}_{12}\\ {p}_{21}\vee {b}_{21}& {p}_{22}\vee {b}_{22}\end{array}& \begin{array}{cc}\cdots & {p}_{1mk}\vee {b}_{1mk}\\ \cdots & {p}_{2mk}\vee {b}_{2mk}\end{array}\\ \begin{array}{cc}\vdots & \vdots \\ {p}_{mk1}{\vee b}_{mk1}& {p}_{mk2}{\vee b}_{mk2}\end{array}& \begin{array}{cc}{p}_{ij}\vee {b}_{ij}& \vdots \\ \cdots & {p}_{mkmk}\vee {b}_{mkmk}\end{array}\end{array}\right\}}_{mk\times mk}=\begin{array}{c}1\\ \begin{array}{c}2\\ \begin{array}{c}\vdots \\ mk\end{array}\end{array}\end{array}{\left\{\begin{array}{cc}\begin{array}{cc}{S}_{11}& {S}_{12}\\ {S}_{21}& {S}_{22}\end{array}& \begin{array}{cc}\cdots & {S}_{1mk}\\ \cdots & {S}_{2mk}\end{array}\\ \begin{array}{cc}\vdots & \vdots \\ {S}_{mk1}& {S}_{mk2}\end{array}& \begin{array}{cc}{s}_{ij}& \vdots \\ \cdots & {S}_{mkmk}\end{array}\end{array}\right\}}_{mk\times mk}$$

In Eq. (4), ${\mathbf{S}}_{ij}^{k}$ indicates that if disassembly task *i* has priority over task *j*, then ${\mathbf{S}}_{ij}=1$; otherwise, ${\mathbf{S}}_{ij}=0$.

According to Eq. (4), when the disassembly task *j* has the highest disassembly priority, it can be performed. Therefore, the feasibility conditions for disassembly task *j* were defined as follows:

5 $$\sum_{i=1}^{mk}{\mathbf{S}}_{ij}^{k}=0$$

The products’ disassembly tasks could be obtained from Eq. (5), and then, ${\mathbf{S}}_{mk}^{k}$ could be updated after disassembly. When ${\mathbf{S}}_{mk}^{k}={\left[0\right]}_{mk\times mk}$, all the disassembly tasks were finished, and the disassembly task hierarchical matrix, ${\mathbf{G}}^{k}$, for the EOL product *k* could be obtained.

**Figure 2:**
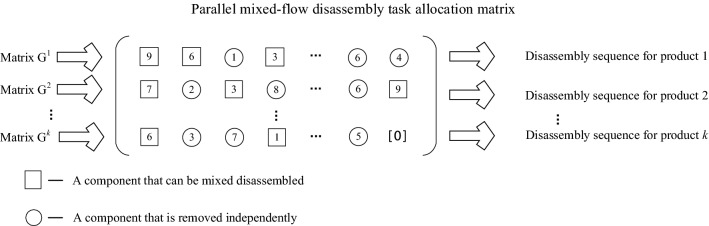
Parallel mixed-flow disassembly task allocation matrix.

The parallel mixed-flow disassembly task allocation matrix, $\mathbf{G}=\left\{{\mathbf{G}}^{1},{\mathbf{G}}^{2},{\mathbf{G}}^{3},\cdots ,{\mathbf{G}}^{k}\right\}$, shown in Fig. 2, could then be obtained from Eqs. (2)–(5).

Considering the uncertainty in the number of parts, during the construction of the disassembly sequence matrix for mixed-flow products, the largest number of parts among *k* products should be taken as the matrix column standard, and the elements of the matrix with insufficient parts among the other products should be filled with 0.

The mathematical model for the PMRDLB problem was formulated utilizing Eqs. (6)–(13).

6 $$\mathrm{min}F=\left({f}_{1},{f}_{2},{f}_{3}\right)$$

7 $${f}_{1}=N$$

8 $${f}_{2}=\sqrt{\sum_{r=1}^{N}{\left(CT-\sum_{l=1}^{k}\sum_{i=1}^{mk}{t}_{il}\times {x}_{ilr}\right)}^{2}/N}$$

9 $${f}_{3}=\frac{\sum_{l=1}^{k}\sum_{i=1}^{mk}\left({L}_{il}\times {P}_{il}\right)}{\sum_{l=1}^{k}\sum_{i=1}^{mk}{L}_{il}}$$

10 $$ \mathop \sum \limits_{i = 1}^{mk} \mathop \sum \limits_{l = 1}^{k} \mathop \sum \limits_{r = 1}^{N} x_{ilr} = 1 $$

11 $$ \mathop {\max }\limits_{{r \in \left\{ {1,2, \cdots ,N} \right\}}} \left\{ {\mathop \sum \limits_{l = 1}^{k} \mathop \sum \limits_{i = 1}^{mk} \left( {x_{ilr} \times t_{il} } \right)} \right\} \le CT $$

12 $$ \frac{{\mathop \sum \nolimits_{l = 1}^{k} \mathop \sum \nolimits_{i = 1}^{mk} \left( {x_{ilr} \times t_{il} } \right)}}{CT} \le N \le \mathop \sum \limits_{l = 1}^{k} \mathop \sum \limits_{i = 1}^{mk} x_{ilr} $$

13 $$ \mathop \sum \limits_{r = 1}^{N} \left( {x_{nlr} \times r} \right) \le \mathop \sum \limits_{r = 1}^{N} \left( {x_{mlr} \times r} \right)\quad n = \left( {1,2,3,L,mk - 1} \right),\quad m = \left\{ {m{|}m = n + 1} \right\} $$

Equations (6)–(9) represent the optimization objects. In these equations, *f*_1_ is the number of parallel mixed-flow disassembly line stations, *f*_2_ is the station equalization rate, and *f*_3_ is the remanufacturing value index, which ensures disassembly of the higher value remanufacturing components as early as possible to avoid secondary-operation damage to the remanufacturing cores. Equation (10) ensures that each disassembly line and disassembly task are assigned only to one station. Equation (11) guarantees that the maximum total disassembly time in each disassembly station does not exceed the beat time, *CT*. Equation (12) represents the workstation number range in the parallel disassembly line. Equation (13) ensures that the priority relationship constraint is met for all of the disassembly tasks during an EOL product’s disassembly.

### PMRDLB problem solution based on the improved NSGA-III

Remanufacturing disassembly line balancing is a multi-objective optimization problem (MOP). The fast, non-dominated genetic algorithm NSGA-III with an elite strategy is characterized by fast operation and a high-precision solution. However, when it is used to solve the PMRDLB problem, its low sorting efficiency and unmatched hierarchical structure for disassembly tasks present significant challenges. Therefore, the NSGA-III algorithm was improved: the chromosome was coded based on the parallel mixed-flow disassembly task assignment matrix, and a non-dominant solution sorting method based on the Pareto rank was developed.

#### Chromosome construction method based on the parallel mixed-flow disassembly task assignment matrix

The multi-variety parallel mixed-flow remanufacturing disassembly line included many different kinds and quantities of EOL products. Therefore, a stratified two-segment chromosome coding method was proposed, as shown in Eq. (14).

14 $${\varvec{c}}{\varvec{o}}{\varvec{d}}{\varvec{e}}=\left(\mathrm{MixedS},\mathrm{FV}\right)=\left(\left(\begin{array}{cccccc}{\mathrm{g}}_{1}^{1}& {\mathrm{g}}_{2}^{1}& \cdots & {\mathrm{g}}_{\mathrm{i}}^{1}& \cdots & {\mathrm{g}}_{\mathrm{m}1}^{1}\\ {\mathrm{g}}_{1}^{2}& {\mathrm{g}}_{2}^{2}& \cdots & {\mathrm{g}}_{\mathrm{i}}^{2}& \cdots & {\mathrm{g}}_{\mathrm{m}2}^{2}\\ \cdots & \cdots & \cdots & \cdots & \cdots & \cdots \\ {\mathrm{g}}_{1}^{\mathrm{k}}& {\mathrm{g}}_{2}^{\mathrm{k}}& \cdots & {\mathrm{g}}_{\mathrm{i}}^{\mathrm{k}}& \cdots & {\mathrm{g}}_{\mathrm{mk}}^{\mathrm{k}}\end{array}\right),\left({f}_{1,}{f}_{2},{f}_{3}\right)\right)$$

In Eq. (14), the first segment, MixedS, represents the disassembly task sequences, and $\mathrm{FV}$ denotes the multi-objective fitness function values. The number of workstations, *f*_1_, the equalization rate, *f*_2_, and the remanufacturing value index, *f*_3_, could be decoded according to Eqs. (7)–(9).

To improve the convergence speed and solution precision of the algorithm, a chromosome construction method, which was based on the parallel mixed-flow disassembly task allocation matrix, was proposed to ensure that all chromosomes were feasible solutions under the constraints of the parallel mixed-flow remanufacturing disassembly line. The method contained four primary steps:

Step 1: According to the disassembly process scheme for EOL products, *k* kinds of disassembly task priority matrices, ${\mathbf{P}}_{m}^{k}$, and hazard mapping matrices, ${\mathbf{B}}_{m}^{k}$, were constructed. The comprehensive priority matrix, ${\mathbf{S}}_{m}^{k}$, was deduced according to Eq. (4). The initial population matrix was defined as Q, and the layered matrix, ${\mathbf{G}}^{k}$, of disassemblable parts of EOL products was defined as a zero matrix.

Step 2: The disassemblable parts were put into the disassembly task hierarchy matrix, **G**^*k*^, and the ${\mathbf{S}}_{m}^{k}$ matrix was simultaneously updated. It was determined whether ${\mathbf{S}}_{m}^{k}$ was a zero matrix. If so, *i* was set to 1 and the method moved to Step 3; otherwise, Step 2 was repeated.

Step 3: The *i*th line in **G**^*k*^ was removed, *pop* gene fragments were randomly generated and stored in **Q**, the *i*th line of **G**^*k*^ was set to 0, and *i* was incrementally increased.

Step 4: If ${\mathbf{G}}^{k}$ was determined to be a zero matrix, **Q** was output; if not, the method returned to Step 3.

**Figure 3:**
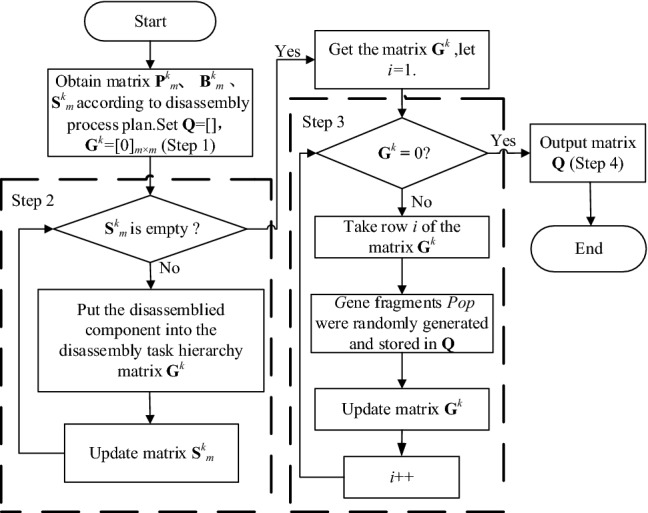
Chromosome acquisition flowchart.

A flowchart for the chromosome construction method is shown in Fig. 3.

#### Chromosome evolutionary rules

The initial population could be determined according to the chromosome acquisition method presented in Fig. 3, and the offspring population would be generated by chromosome cross and mutation operations. Furthermore, the structural reference points were established based on the Pareto rank.

**Figure 4:**
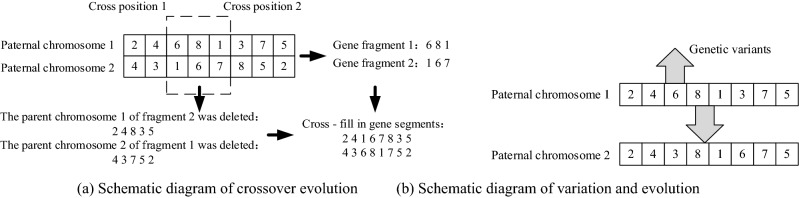
Schematic crossover and variation diagram.

1. *Cross and mutation operations* Two paternal chromosomes, 1 and 2, were randomly selected from the initial population, and two cross sites, 1 and 2, on the paternal chromosomes were randomly determined. The gene fragments between the two cross sites were called fragments 1 and 2, and the gene containing fragment 2 on paternal chromosome 1 was deleted. The gene containing fragment 1 on paternal chromosome 2 was also deleted, and fragment 2 was inserted into paternal chromosome 1 according to the cross positions 1 and 2 to form a new chromosome 1. Fragment 1 was inserted into paternal chromosome 2 according to the cross positions 1 and 2 to form a new chromosome 2. Two mutation sites, 1 and 2, were determined randomly, and genes were exchanged at these sites on the new chromosomes to form offspring chromosomes, 1 and 2. The schematic chromosome crossover and variation diagram is shown in Fig. 4a. The selected chromosome genes mutated to produce new chromosomes, as shown in Fig. 4b.
2. *Non-dominated ranking* During the comparison process, if *R*1 and *R*2 fulfilled ${f}_{i}(R1)\le {f}_{i}(R2)(\forall i\in (\text{1,} \, \text{2,} \, {3}))$, then *R*1 dominated *R*2. If *R1* was not dominated by other vectors, then *R*1 was the Pareto solution.The dominant relationship was determined by a Pareto comparison of the objective function values of *R*1 and *R*2. When *R1* dominated *R*2, the Pareto level of *R*1 was 1 and was denoted as Pareto 1. Similarly, the chromosomes’ Pareto levels could also be obtained.The (*s* + 1)th generation was a combination of the parent population and the progeny population and was sorted according to the chromosomes’ Pareto ranks.

#### Generation of the structured reference points

The NSGA-III ensures solution diversity by using a predefined set of reference points, which can be defined in a structured manner^19^. Reference points were uniformly distributed points in the PMRDLB model’s solution space, which was in an (*M* − 1) dimensional hyperplane, where *M* is the dimension of the target space, namely, the number of optimized targets. If each target was divided into *H* parts, there were four primary reference point generation steps:

Step 1: The number of reference points, *H*, was determined using the following equation:

15 $$H={\mathrm{C}}_{M+p-1}^{p}$$

where the *p*th coordinate axis was divided into several parts.

Step 2: The extremum point of the objective function was determined. The target value was very large, and the target value of the individual corresponded to the small points on other target values. The minimum value of the three objective functions in this study was $\overline{Z }=\left({Z}_{1}^{\mathrm{min}},{Z}_{2}^{\mathrm{min}},{Z}_{3}^{\mathrm{min}}\right)$; so, the extreme point was solved according to Eq. (16).

16 $${f}_{i}^{^{\prime}}\left({\text{x}}\right)={f}_{i}\left({\text{x}}\right)-{z}_{i}^{\mathrm{min}}$$

Step 3: The distances between the target point and the reference points on extract chromosomes were calculated, and the selected chromosomes were added to the next generation population.

Step 4: Steps 2 and 3 were repeated until the population size was consistent.

#### PMRDLB model optimization process

Optimizing the PMRDLB model was performed to achieve a reasonable allocation of disassembly tasks at the stations. The optimization process included five primary steps: data preparation, initial population acquisition, non-dominated ranking based on the Pareto level, structured reference point generation, and optimal solution output.

Step 1: In the data preparation stage, the disassembly process plan for EOL products was analyzed to obtain the comprehensive priority relationship matrix and define and initialize the parameters, such as the population size (*pop)*, the beat time (*CT)*, and the number of iterations (*Gen)*.

Step 2: The disassembly task allocation matrix was obtained, the objective function value was calculated, the chromosomes were generated, and the initial population was established.

Step 3: The offspring population was generated by cross and mutation operations. The parent and child populations were combined, and the chromosomes’ Pareto ranks were determined.

Step 4: Next-generation chromosomes were extracted based on the structured reference points.

Step 5: The optimal non-dominated solution set was obtained.

**Figure 5:**
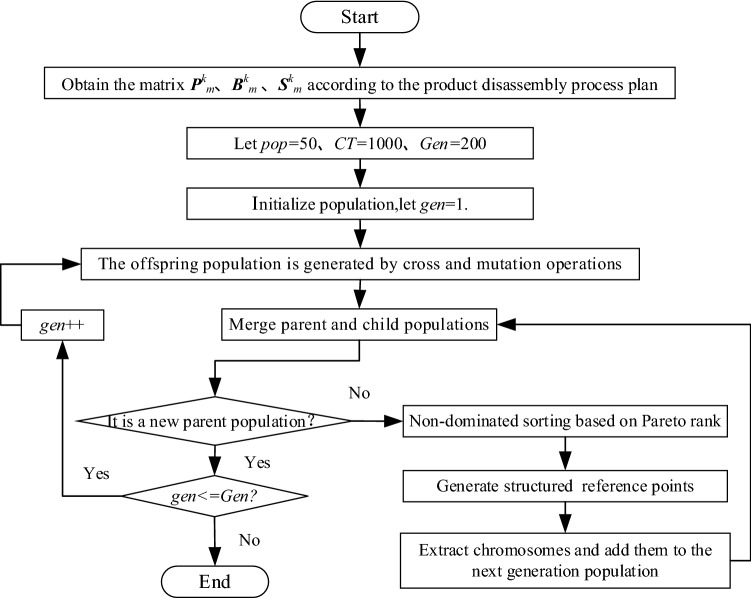
Flowchart for the PMRDLB problem solution process.

The solution process for the PMRDLB problem, which was based on the improved NSGA-III algorithm, is shown in Fig. 5.

## Results and discussion

To verify the feasibility and effectiveness of the proposed method, a 34-component engine^32^ and a 37-component Passat B5 engine^33^ were selected for a case study. The remanufacturing values were generated by random numbers ranging from 0 to 100, and the component information is presented in Table 3.

**Table 3:** Component information for the two engine types.

34 Task	Name	Disassembly time (s)	Remanufacturing value (￥)	Harmfulness	37 Task	Name	Disassembly time (s)	Remanufacturing value (￥)	Harmfulness
1	Alternator support bracket	38	5	No	1	Igniter	164	82	Yes
2	Alternator	23	65	Yes	2	Valve cover	96	91	No
3	Drive belt	30	0	No	3	Camshaft	326	12	No
4	Water pump pulley	56	15	No	4	Valve assembly	251	92	No
5	Special washers	161	35	No	5	Timing belt	0	63	No
6	Crankshaft Pulley	10	40	No	6	Camshaft drive wheel	49	9	No
7	Pulley	12	45	No	7	Timing belt tensioning wheel	56	28	No
8	Oil level indicator	12	65	No	8	Timing belt toothed belt wheel	40	55	No
9	Synchronous band cover	70	8	No	9	Crankshaft gear	156	96	No
10	Synchronous belt lower cover	140	8	No	10	Intake manifold	163	97	Yes
11	Timing belt	45	0	No	11	Exhaust manifold	145	15	No
12	Belt tension spring	34	60	No	12	Air cleaner	93	98	Yes
13	Tensioner	34	70	No	13	Intake pipe	54	96	No
14	Crankshaft sprocket flange	70	30	No	14	Turbocharger	56	49	No
15	Crankshaft sprocket	10	45	No	15	Supercharger flywheel	86	80	No
16	Water pump	56	95	Yes	16	Supercharger belt	0	14	No
17	Rocker cover and gasket	11	50	No	17	Supercharger belt tensioning wheel	72	42	No
18	Intake pipe	180	40	No	18	Supercharger pump wheel	94	92	No
19	Exhaust pipe	144	40	No	19	Engine support frame	76	80	No
20	Cylinder distributor camshaft valve	730	95	No	20	Cylinder block	240	96	No
21	Oil filter	60	65	No	21	Connecting Rod	162	66	No
22	Oil receiver	126	40	No	22	Large tile	76	3	No
23	Oil screen	38	40	No	23	Small tile	74	85	No
24	Oil cap	10	25	No	24	connecting rod cover	153	94	No
25	Front case	84	15	No	25	Crankshaft main bearing cap	72	68	No
26	Oil pump	54	95	No	26	Crankshaft	312	76	No
27	Pistons, connecting rod	24	30	No	27	Oil pump	50	75	No
28	The connecting rod cup	24	30	No	28	Oil pump chain	175	39	No
29	Flywheel	675	45	No	29	Intake camshaft lock block	115	66	No
30	Thick steel plate	44	50	No	30	Vent camshaft lock block	115	17	No
31	Engine bell housing	36	65	No	31	Oil pan	123	71	No
32	Oil seal cover	63	30	No	32	Transmission assembly	183	3	No
33	Rear oil seal	30	25	No	33	Clutch flywheel	72	27	No
34	Crankshaft	530	55	No	34	Clutch pressure plate	65	4	No
–	–	–	–	–	35	Clutch cover	82	9	No
–	–	–	–	–	36	air cylinder	265	83	No
–	–	–	–	–	37	Clutch disc	12	70	No

### Calculations of structural similarity between the two engines

The 34-component engine and the 37-component Passat B5 engine were two different kinds of engines with different uses. A similarity analysis was conducted on the two engines using expert judgment, and the results are presented in Table 4.

**Table 4:** Results of a similarity analysis of the two engines.

Criteria	Task 34 engine parts	Task 37 Passat B5 engine parts	Similar structure
Structural similarity	Drive belt, water pump pulley, crankshaft pulley, belt pulley, synchronous belt cover, synchronous belt cover, synchronous belt, belt tension spring, belt tensioner	Timing belt, Timing belt tensioning wheel, Timing belt toothed belt wheel, Supercharger belt, Supercharger belt tensioning wheel	Belt structure
	Piston, rod, rod cup	Connecting rod, Large tile, Small tile, Connecting rod cover	Connecting rod construction
	Crankshaft, camshaft	Crankshaft main bearing cover, Crankshaft, Intake Camshaft lock block, Outlet camshaft lock block, Camshaft	Shafting structure
	Air cylinder, intake pipe, exhaust pipe	Cylinder, Cylinder block, Intake pipe, Outlet manifold, Intake manifold, Valve assembly, Valve cover	Cylinder construction
	Crankshaft sprocket flange, crankshaft sprocket	Oil pump chain	Sprocket structure
	Back oil seal, sealing oil cap, oil pump, oil cap, oil filter net, oil pan, oil filter, oil level indicator	Oil pump, oil sump	Oil pump structure

According to Eq. (1), the product similarity, ${\lambda }_{pro}=\frac{\bigcap \left\{{a}_{1}^{1},{a}_{1}^{2},{a}_{2}^{1},{a}_{2}^{2},{a}_{3}^{1},{a}_{3}^{2}\cdots ,{a}_{m1}^{1},{a}_{m2}^{2}\right\}}{\bigcup \left\{{a}_{1}^{1},{a}_{1}^{2},{a}_{2}^{1},{a}_{2}^{2},{a}_{3}^{1},{a}_{3}^{2}\cdots ,{a}_{m1}^{1},{a}_{m2}^{2}\right\}}=\frac{\mathbf{S}}{{\mathbf{P}}_{1}{\mathbf{P}}_{2}}=\frac{51}{{71}}\text{=} \, \text{0.71}$, was obtained, which satisfied ${\lambda }_{pro}\ge 0.7$. Therefore, the mixed-flow disassembly line operation could be conducted.

### Problem-solving

The computer used in the case study was an Intel(R) Core I5-6200U CPU with 2.30 GHz and 12 GB RAM. The PMRDLB prototype system was developed using a professional edition of MATLAB R2016a in Windows 10.

After building the disassembly task allocation matrix according to Eqs. (2)–(6), the number of iterations and the population size were set to *Gen* = 200 and *pop* = 50, respectively. The disassembly task time, 730 s, was the total task time of the maximum disassembly workstation for the 34-component engine. Therefore, the beat time was *CT* ≥ 730 s, and five optimal disassembly line configuration schemes were obtained by 20 experiments, as shown in Table 5.

**Table 5:** Optimal PMRDLB scheme.

Plan	Plan 1		Plan 2		Plan 3		Plan 4		Plan 5	
Engine name	Task 34	Task 37	Task 34	Task 37	Task 34	Task 37	Task 34	Task 37	Task 34	Task 37
Disassembly task number	21	1	22	1	18	13	3	7	3	1
	18	7	21	7	21	35	8	19	18	7
	8	13	18	19	22	19	9	17	19	17
	3	17	3	35	19	7	18	35	8	13
	9	19	8	13	9	1	21	13	22	35
	19	35	9	17	8	17	22	1	9	19
	22	5	19	16	3	5	19	2	21	32
	4	12	1	5	4	12	1	12	4	16
	1	32	17	12	5	32	5	16	23	5
	5	16	4	2	1	2	4	32	1	2
	23	2	5	32	17	16	23	5	5	12
	17	8	23	10	23	8	17	33	17	30
	2	10	2	33	16	30	2	30	16	33
	16	30	16	8	2	10	16	29	2	10
	6	11	6	30	6	29	6	11	6	11
	7	15	7	11	7	15	7	8	7	6
	10	29	10	6	10	6	10	10	10	8
	12	6	12	9	12	9	12	15	12	9
	11	9	13	29	13	33	13	6	11	29
	13	33	11	15	11	11	11	9	13	15
	14	18	14	18	14	18	14	18	15	18
	15	14	15	14	15	14	15	14	14	14
	25	34	25	34	24	3	25	3	20	34
	24	3	20	3	20	34	24	34	25	3
	20	4	24	37	25	4	20	4	24	4
	26	37	27	4	26	37	28	37	26	37
	27	31	26	31	27	31	26	31	27	31
	28	28	28	28	28	28	27	28	28	25
	29	25	29	24	29	24	29	25	29	28
	34	24	30	25	34	25	34	24	30	24
	30	27	34	22	30	27	30	27	34	23
	31	23	31	23	31	22	31	23	31	22
	32	22	32	27	32	23	32	22	32	27
	33	21	33	21	33	21	33	21	33	21
	–	36	–	36	–	36	–	36	–	36
	–	26	–	26	–	26	–	26	–	26
	–	20	–	20	–	20	–	20	–	20
Number of workstations *f*_1_	*f*_1_ = 9		*f*_1_ = 10		*f*_1_ = 10		*f*_1_ = 11		*f*_1_ = 9	
Equilibrium rate *f*_2_	*f*_2_ = 33.50		*f*_2_ = 52.30		*f*_2_ = 55.40		*f*_2_ = 65.30		*f*_2_ = 45.8	
Remanufacturing value index *f*_3_	*f*_3_ = 98.59		*f*_3_ = 99.52		*f*_3_ = 97.56		*f*_3_ = 94.32		*f*_3_ = 97.35	

**Figure 6:**
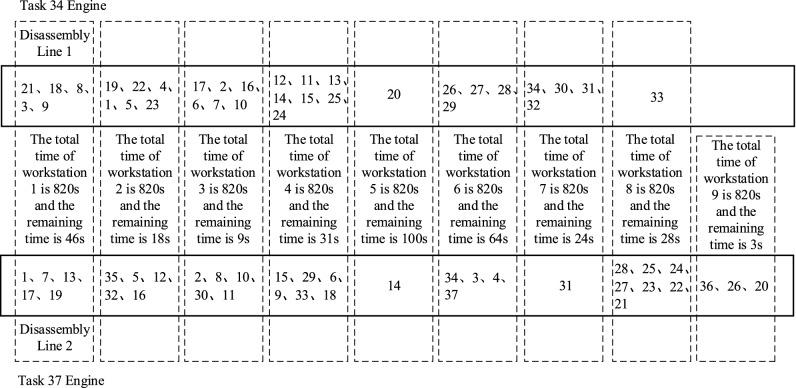
Parallel mixed-flow disassembly line layout for plan 1.

Taking plan 1 as an example, the disassembly task assignment results are shown in Fig. 6.

To verify the effectiveness of the model and method proposed in this paper, the objective function values *f*_1_, *f*_2_, and *f*_3_ of the mixed-flow disassembly line for task 1 under different layout forms were compared, and the results are shown in Table 6.

**Table 6:** Comparison of solution results for remanufacturing disassembly lines with different layouts.

Layout forms	Number of workstations *f*_1_	Workstation equalization rate *f*_2_	Remanufacturing value index *f*_3_
Parallel mixed-flow	9	33.50	98.59
Straight line mixed-flow	11	101.66	1959.43
U type mixed-flow	10	76.34	980.36

Table 6 shows that, compared with other layout forms, the parallel mixed-flow remanufacturing disassembly line improved the disassembly efficiency and had obvious advantages for solving the multi-variety EOL product disassembly problem. The disassembly line model for parallel mixed-flow remanufacturing proposed in this paper overcame the above shortcomings and solved the problem when there were many kinds of recovered waste products and the number of parts was uncertain. Experimental results showed that the method was feasible and effective.

## Conclusions

There are many types of EOL products in remanufacturing disassembly lines, and the number of components is often uncertain. To solve this problem, a PMRDLB optimization model was proposed in this paper, and the NSGA-III algorithm was improved. Two engine cases were studied to verify the validity of the proposed model and method.

The method had three primary highlights:

1. In view of the uncertain characteristics of multi-variety products in remanufacturing disassembly lines, a parallel mixed-flow remanufacturing disassembly line layout was designed. It not only made reasonable use of space but also improved the efficiency of parallel mixed-flow disassembly for multi-variety products.
2. A construction method for the mixed-flow product disassembly task allocation matrix was proposed, which overcame the difficulties of model construction and low computational efficiency caused by the traditional mixed disassembly line in which multiple products were regarded as a single imaginary mixed product.
3. The NSGA-III algorithm was improved to solve the PMRDLB problem. A stratified two-segment chromosome coding method was adopted to ensure that all solutions were feasible. This method also improved the optimization efficiency.

## Data Availability

The datasets used and/or analysed during the current study available from the corresponding author on reasonable request.
